# Navigating the Minefield of Computational Toxicology and Informatics: Looking Back and Charting a New Horizon

**DOI:** 10.3389/ftox.2020.00002

**Published:** 2020-06-25

**Authors:** Grace Patlewicz

**Affiliations:** Independent Researcher, Durham, NC, United States

**Keywords:** informatics, read-across, data science, computational toxicology, (Q)SAR

## Introduction

As we enter 2020, it is worth looking back at the development and progression of the computational toxicology discipline, how it has evolved and what some opportunities might be going forward. Computational toxicology stands poised to broadly and directly inform chemical safety assessment, and as such, the demands of computational toxicology are growing due to international regulatory needs. Critical to increasing scientific confidence in the use of computational toxicology approaches in applied toxicology decision-making will be: (1) transparency and reproducibility in the underlying data and data analysis approaches utilized; (2) accessibility of information to evaluate the fitness of the computational toxicology approach for a particular problem; and (3) sharing of ideas and approaches internationally. Herein the progress in applied computational toxicology is considered, with a call for additional research to continue this rapid advancement.

## Early Computational Toxicology: Application of Quantitative Structure Activity Relationships [(Q)SARs]

A quarter of century ago, the field of computational toxicology might simply have been summarized as the intersection of three scientific domains: toxicology, chemistry, and statistics, packaged in predictive models such as SAR and QSAR models, collectively referred to as (Quantitative) Structure Activity Relationships [(Q)SARs]. (Q)SARs are theoretical models that can be used to predict in a quantitative (e.g., potency) or qualitative manner (e.g., active/inactive) the physicochemical, biological [e.g., an (eco)toxicological endpoint] and environmental fate properties of compounds from the knowledge of their chemical structure (Worth et al., 2005). A SAR is a (qualitative) association between a chemical substructure and the potential of a chemical containing the substructure to exhibit a certain biological effect. The classic example of a SAR was the supramolecule published by Ashby and Tennant (1988) which related chemical structure moieties to genotoxic carcinogenicity. Typical toxicity endpoints under study were those with a greater preponderance of data such as the Ames test for bacterial mutagenicity (see Benigni and Bossa, 2019 for a recent review), or the fathead minnow fish acute lethality test (Adhikari and Mishra, 2018). Physicochemical properties such water solubility, octanol-water partition coefficient (LogKow) were also modeled. The algorithms underpinning these predictive models (QSAR) tended not to be overly complex mainly because the data volume was usually limited, “big” data was confined to a few hundred data points and typically far less. The algorithms used to develop QSARs relied upon conventional statistical approaches such as linear regression or logistic regression, in part because the data volume did not merit more complex models, and in part since the models being developed relied on a limited set of descriptors that could be readily computed and interpreted relative to the property being modeled. Indeed, many fish toxicity models and Ames models relied upon LogKow as the main determining factor. Computing descriptors for chemicals was mainly dependent on commercial software within specific QSAR modeling platforms, e.g., TSAR from Oxford Molecular, Biovia's QSAR Workbench (https://www.3dsbiovia.com/products/collaborative-science/biovia-qsar-workbench/). The types of QSAR models for toxicity were often “local,” i.e., based on defined mechanisms or chemical classes. The exception tended to be for physicochemical parameters where models were categorized as “global,” i.e., heterogenous training datasets comprising a diversity in chemical structure.

The underlying principle within this framework was that the toxicity (property) being predicted was a function of chemical structure. The notion of similarity where similar chemicals were expected to cause similar toxicities also formed the complementary basis around the concept of read-across (Patlewicz et al., 2018) as well as Thresholds for Toxicological Concern (TTC) approaches (Kroes et al., 2004).

The application of these models was also limited, usually in providing preliminary indications of activity rather than in lieu of additional empirical data. The toxicity would be characterized by a single summary endpoint e.g., point of departure such as a No Adverse Effect Level (Concentration) [NOAEL(C)] and usually a single value for a given substance. The concept of reproducibility of the test method was not a major consideration, since studies tended not be repeated due to cost, animal use, and time constraints.

## Evolving Regulatory Landscape for (Q)SARs in Application

In the late 1990s, there started to be a need to make predictions for a broader coverage of chemicals beyond the smaller datasets that underpinned the mechanistic chemical class type QSAR models to date. The shift was partly driven by interest in improvements to quantitative descriptions of chemical structure for toxicity prediction, and the availability of computing power.

Decision contexts were also changing and provided an additional impetus for new model development. Two main drivers were influencing this change: the need for non-animal alternatives, largely prompted the EU Cosmetics Regulation (European Commission, 2009) and the EU Chemicals legislation known as REACH (European Commission, 2006). REACH in particular had a profound effect in the development, evaluation, and application of QSARs, primarily since the decision context was to use QSAR predictions as supporting information in the construct of an Integrated Approaches to Testing and Assessment (IATA) (Tollefsen et al., 2014) and/or in lieu of new experimental testing. In the run up to REACH coming into force, there was a concerted effort to characterize a framework to facilitate the use of (Q)SARs for regulatory purposes (Cronin et al., 2003a,b). This culminated in the formulation of the OECD QSAR principles for validation namely: a defined endpoint, unambiguous algorithm, appropriate measures of predictivity (e.g., external validation), goodness of fit (e.g., cross-validation), an applicability domain and mechanistic interpretation if possible (OECD, 2004, 2007; Patlewicz et al., 2016). The QSAR Validation principles largely provided the impetus to develop new approaches to characterize the applicability domain of models (Netzeva et al., 2005; Nikolova-Jeliazkova and Jaworska, 2005) as well as consider integration of models e.g., consensus models (e.g., Votano et al., 2004). In the development of Frontiers in Toxicology: Computational Toxicology and Informatics, a focus on the validation principles and their applicability to (Q)SARs and beyond is a component of advancing the scientific confidence in using these approaches in applied decision-making.

## Broadening Computational Toxicology to a Strategic *in silico* and *in vitro* Approach as Supported by Informatics

At the same time, the NRC report was published (NRC, 2007) which outlined the change in how toxicity testing could be undertaken. Subsequent reports on computational methodology for exposure (NRC, 2012) and risk assessment (NRC, 2017) have broadened the call. The NRC reports, together with the synergism of increased computing resources, increased access to laboratory automation for toxicology, and development of methodologies that efficiently generated large volumes of data, generated a disruptive change in the field and an expansion of what computational toxicology represented. Instead of summarizing toxicity on the basis of traditional toxicity tests, a shift was proposed to predict genotoxic vs. non-genotoxic substances, and then to have *in vitro* bioactivity and predicted exposure define a bioactivity:exposure ratio, which would inform the need for models of greater biological complexity (Thomas et al., 2013, 2019). This shift is dependent on high throughput and high content screening methods (HTS/HCS), including high throughput transcriptomics (HTTr) and high throughput phenotypic profiling (HTTP) of cellular morphology (Harrill et al., 2019; Thomas et al., 2019; Nyffeler et al., 2020).

The data needed for a rapid, high-throughput safety assessment requires application of a range of computational approaches for data analysis, data storage, and *in silico* predictive modeling. These challenges are directly identified in the title of this journal as the necessary “informatics” component of realizing computational toxicology for safety assessment. How to meet these informatic challenges is the subject of ongoing research as the volume and variety of data require tools for large scale data processing, databasing and informatics for single-dimension and multi-dimensional datasets, visualization for heterogeneous information, demonstrating reproducibility, and quality control, and perhaps most challenging, for interpretation and communication in the appropriate format and context for chemical safety assessment. Many aspects of the vision articulated by the initial NRC report have been realized in preliminary form by the ToxCast (Kavlock et al., 2012) and Tox21 research programs (Tice et al., 2013; Thomas et al., 2018) which have generated publicly available HTS data on thousands of chemicals. In addition to the data generated, data processing pipelines have been developed (Hsieh et al., 2015; Filer et al., 2017) and many different models continue to be derived using the data, including those designed to understand mode-of-action (e.g., Shah et al., 2011; Judson et al., 2015; Kleinstreuer et al., 2016; Saili et al., 2019;) as well as models that use HTS data as descriptors or training information for (Q)SARs (e.g., Liu et al., 2015; Mansouri et al., 2016). The informatic needs of data driven predictive modeling, and how to standardize and openly transmit these models, is a clear need in the field. Recent progress in advancing computational toxicology and the associated challenges were discussed in Ciallela and Zhu (2019). Noteworthy examples of recent data driven models include those for acute oral toxicity (Russo et al., 2019) and liver toxicity (Zhao et al., 2020).

The databasing and informatic challenges for computational toxicology also have a legacy component: to bolster scientific confidence and for fit-for-purpose evaluations, the use of *in vivo* animal study data and any available *in vivo* human data have been important in the early application of computational toxicology as replacements or alternatives to existing approaches (Kleinstreuer et al., 2016, uterotrophic database; Hoffmann et al., 2018, LLNA database; Watford et al., 2019b; ToxRefDBv2). To enable quantitative comparisons of dose in animals or humans, internal exposures as modeled using HT toxicokinetic data and modeling (Wetmore et al., 2012; Pearce et al., 2017; Wambaugh et al., 2018) have been developed to support *in vitro* to *in vivo* extrapolation (IVIVE). Examples of how IVIVE has enabled greater utilization of HTS data for safety assessment are discussed in more detail in Thomas et al. (2019); Paul Friedman et al. (2020).

## Current State of Computational Toxicology and Informatics

### The Rising Need of Informatics and Data Engineering

The validation principles of QSARs are perhaps still relevant today but in providing a framework to make explicit the provenance of the data, how it has been processed, the assumptions made, and the transparency and reproducibility of any models derived (Patlewicz et al., 2015). The three pillars of statistics, toxicology, and chemistry have since extended, in part due to the demand to make rapid decisions (Judson et al., 2010), with greater transparency. Perhaps the term “Data Science” now better captures the skill sets and needs encompassed in computational toxicology. Thus, computational toxicology considers the disciplines of toxicology, chemistry, and statistics, but also a number of front-end data science techniques relying on programming skills to facilitate data acquisition, data processing, storage and retrieval, data manipulation and interpretation, and beyond traditional statistics, other machine learning and deep learning techniques. The wealth of open source tools has also facilitated the change in the skills and approaches now applied. Commercial bespoke tools are being somewhat superseded by open source libraries developed on top of programming languages such as R and python. A skill set that so far has not been a strong focus as yet is that of data engineer, the backend of data science: models developed need to be deployed, and for reproducible models, a different set of considerations regarding versioning of models, their underlying inputs, and algorithms e.g., docker containers.

### Evaluating Fit-For-Purpose Utility

The variety and volume of data now being generated and analyzed has also raised challenging questions for the “legacy” or existing *in vivo* data available: the level of curation, study reproducibility, and how these data may be used to benchmark new approach methodologies are all of high interest (Pham et al., 2019) Using *in vivo* study data to benchmark the performance of or directly train new approach methodologies for human or ecological health assessment should include some evaluation of how variable the *in vivo* study data may have been. Fit-for-purpose evaluations require not only acquisition of and curation of reference data and meaningful assessments of variability and uncertainty, but also efforts to increase data interoperability (Watford et al., 2019a). That requires ontologies in data storage and domain knowledge, as well as standards that permit sharing and exchange of data and models. Another consideration is the fact that these new data stream technologies are evergreen and in constant state of evolution and improvement. Evaluation of the fitness of these information needs to be flexible to deal with the changes in the methods of a specific technology and increased understanding of method performance using a large number of substances (Judson et al., 2018; Ciallela and Zhu, 2019).

The concept of an “applicability domain” is central in an evaluation of fit-for-purpose use of computational toxicology approaches, taking on an extended meaning as we want to understand the relevance of the model and when it can be applied and the extent to which it can be used to forecast other substances and to what extent there is confidence for that to occur. The uncertainties associated with the prediction need to be clearly specified and linked back to the decision context and purpose intended. The appropriate measures of fit and predictivity remain important considerations. What are the steps and procedures that were applied during the model building phases, including selection of approach, cross validation, performance metrics, and hyperparameter optimization. Before final model evaluation and application to new data (prediction), consideration of how to deploy a model should include a plan for ensuring reproducibility.

### And Now: A Call for Research and Action Using the QSAR Validation Principles as a Guide

Clearly the landscape of computational toxicology has significantly evolved in the last two decades and much progress has already been made. Notable examples include data challenges that have been organized by NCATS (e.g., Huang and Xia, 2017) and NICEATM (e.g. Kleinstreuer et al., 2018). Most progress has been realized for individual discrete substances but major areas of effort that remain to be tackled include: (1) the challenge of big data itself e.g., how to fit for large datasets (Ciallela and Zhu, 2019) (2) difficult substances to test in existing HTS systems, e.g., volatiles, insoluble in solvents; (3) mixtures–to date progress has been made on developing individual models but less focus on ensemble models; (4) wide implementation of cloud resources for data accessibility and data processing; (5) metabolism and degradation aspects in inferring effects of parent chemicals; (6) the use of unbalanced datasets in model training and development; (7) predicting dose response in conjunction with effects rather than extracting a summary metric from a study (Moran et al., 2019); (8) mining and extraction of insights from unstructured literature data; (9) standardized application of epidemiology; and likely a myriad of other challenges yet to be identified.

In many respects the (Q)SAR validation principles from 2004 remain relevant. The defined endpoint of a model or new approach methodology, the purpose and the goal of the model, and its basis need to be specified, albeit characterized differently to meet the requirements of 2020 and beyond. “The unambiguous nature of the algorithm” reads now as a call for increased reproducibility of the methodology and the approach. Are the assumptions of the modeling approach and the underlying data clearly specified? What are the data processing steps taken? How has the data been generated, summarized, and stored? These and other considerations should feature prominently in *Computational Toxicology and Informatics*.

## Author Contributions

GP prepared and wrote this article.

## Conflict of Interest

The author declares that the research was conducted in the absence of any commercial or financial relationships that could be construed as a potential conflict of interest.
